# Assessing Thresholds for Nerve Activation and Action Potential Block Using a Multielectrode Array to Minimize External Stimulation

**DOI:** 10.3390/bioengineering12040372

**Published:** 2025-04-01

**Authors:** Ashutosh Mishra, R. P. Joshi

**Affiliations:** 1 Department of Applied Science (Bioengineering), IIIT-Allahabad, Prayagraj 21101, India; am@iiita.ac.in; 2 Department of Electrical and Computer Engineering, Texas Tech University, Lubbock, TX 79409, USA

**Keywords:** action potential block, multielectrode stimulation, threshold current, unmyelinated nerve

## Abstract

Devices based on electrical stimulation are in common use for a variety of therapeutic bio-applications, including, but not limited to, neuro-prosthetics, pain management or even in situ local anesthetic modalities. Many require the use of multielectrode systems to selectively activate a group of nerves. In this context, a modeling study is carried out to probe some of the details of nerve activation resulting from multielectrode excitation. In particular, aspects such as threshold stimulus currents, their variation with the number of electrodes used, dependence on nerve radii, and the possibility of blocking an action potential (AP) have been quantitatively analyzed. The injection currents needed to initiate an AP are shown to decrease as the number of stimulating electrodes increases. It is also demonstrated that blocking AP propagation in a nerve segment could be achieved more efficiently at lower magnitudes of the interruption signal if more electrodes in an electrical excitation array were to be used. This result is important and would have practical relevance since the lower intensity external signals indicate a safer and more reliable approach to both AP launches and possible AP blockages.

## 1. Introduction

Interest in electrical stimulation as a therapeutic modality in bioengineering applications has increased dramatically over the past few decades. Advances in technology, material science, and superior electrical delivery systems have been partly responsible for many of the successes. Therefore, as a first step, the various biomedical applications of electrically driven technologies and therapeutic concepts are briefly discussed. In this context, the use of multielectrode arrays for electrical bio-stimulation is also mentioned. This collectively provides the necessary background and serves as a natural segue into the need to adequately model electrical nerve stimulation by multielectrode arrays.

In recent years, external electrical stimulation has increasingly been applied to achieve numerous therapeutic benefits in the bioengineering field. For example, such stimulation has been used to activate muscles, with applications in neurophysiology, neuromuscular therapies, clinical research, and fatigue reduction [1,2,3,4,5]. It offers a means to promote coordinated movements such as walking, and in cases of spinal cord injury or stroke, it helps improve circulation in paralyzed limbs [6]. Examples of unmyelinated nerves are the C-type nerve fibers (such as postganglionic fibers) that are involved in the afferent transfer of temperature, burning pain and itching sensation from the peripheral regions to the spinal dorsal horn. Other reported applications of electrical bio-stimulation have included efforts at blocking the propagation of action potentials (AP) and inhibitions in muscle force [7,8]. Both external DC and high-frequency AC stimulations have been shown to cause such blockage of neural conduction [9,10]. Related modeling and simulation work in this context has also been reported [11,12], with the possibility of using ultrashort electrical pulses for arresting nerve conduction based on electroporation [13,14].

Applications of electrical signals for biomedicine have also been demonstrated in a class of therapies targeted towards autonomic nerves for purposes of altering neural activity for restoring functionality [15,16,17]. In this regard, implantable electrode arrays are being widely used for both clinical studies and in practical therapies. They have also provided advanced understanding of the nervous system in the context of basic neuroscience research. Implantable electronic devices are being utilized to record neurological signals and stimulate nerves to restore lost function. Additionally, implanting electrodes has also been a basis in aiding the restoration of hearing, sight, and balance [18,19,20].

Furthermore, electrical stimulation has been promising in enhancing critical cognitive functions, such as memory. For example, deep brain stimulation has been successful in the treatment of movement disorders, Parkinson’s disease [21], obsessive-compulsive disorders [22], epilepsy [23], and depression [24]. Electrical stimulation has even been an effective therapy for promoting nerve regeneration and is thought to arise from the increased expression of the Brain-Derived Neurotrophic Factor (BDNF) [25]. These treatments all use electrical stimulation to evoke APs in targeted nerves.

Neuromuscular control by artificial electrical stimulation usually requires the use of multielectrode systems that selectively stimulate a group of nerves without exciting all the nerve fibers. For functional neuromuscular stimulation, such selectivity of nerve subgroups in myelinated motor nerves is very desirable. In addition, developing multielectrode systems that have reliable, reproducible, and selective muscle force recruitment characteristics is important in realizing practical neuro-prosthetic systems. In this context, it is germane to probe some of the details of nerve activation resulting from multielectrode excitation. In particular, aspects such as the threshold stimulus current magnitudes, its variation with the number of electrodes used, the effect on nerves of different radii, and the possibility of blocking an AP all merit quantitative analyses. However, to the best of our knowledge, these aspects have not been examined. In this study, we focus on concurrent stimulation of multiple-nodes (i.e., an expanded stimulus zone) while exploring nerve activation, and also probe action potential blockage by the concurrent application of an interruption signal at multiple nodes. To the best of our knowledge, a comprehensive study of multi-node stimulation has not been previously reported.

In this contribution, the thresholds for both nerve activation and AP block with a multielectrode array are assessed based on numerical modeling. This constitutes a simple first step for applying multielectrode stimulation to complex biological systems or to assess whole-body responses. To our knowledge, there exists a gap in the exploration of methods for expanding the stimulation zone in nerve stimulation, which represents the novelty of this contribution. Our hypothesis is that increasing the number of stimulating electrodes would reduce the required injection current for both AP initiation and blocking. As a result, the safety and reliability of the bio-system would be enhanced due to a lowering of the requisite signal intensity. These findings would have practical implications for neurostimulation therapies, neuromuscular control, and bioelectronic medicine, offering a pathway to optimized stimulation strategies. The above hypothesis is verified through our modeling, and it is demonstrated that both launching and arresting AP propagation in a nerve segment can be achieved by applying lower electrical signals. Thus, it also answers the following research question: is it possible to lower the stimulus energy required for nerve activation and block by expanding the application zone of the said stimulus energy? Here, particular emphasis is also on bio-response dependencies relating to the number of stimulating electrodes. In the absence of human testing, such computer-based analyses provide a quick, safe and effective way to make predictions, with the results ultimately leading the way towards optimized treatments. The calculations of nerve simulation discussed here are based on the well-known Hodgkin–Huxley (HH) model [26]. While this model is quite suitable for most studies pertaining to unmyelinated nerves and also readily extends to myelinated nerves through simple changes of a few parameters [27], it does seem to exhibit some computational failures when modeling nerves of diameters under 1 µm. However, it does readily adapt to multielectrode stimulus scenarios, given that it employs the Kirchoff’s current law at each discretized element of the nerve where a specific stimulus current can be injected. We would also like to point out that since the rate parameters (α and β) for the different gating functions and the gating functions themselves are only dependent on the nodal transmembrane potential, they do not require any special or additional treatment with multielectrode stimulus, as there is no traveling wave. However, extensions and the inclusion of other details, such as stochastic noise within the HH framework, could additionally be incorporated based on methods that invoke multi-state Markov process descriptions [28] or other numerical approaches that include fluctuations in the states of discrete ion channel populations [29,30,31,32]. Our group has recently reported on a simple extension of the HH model to include stochastic noise [32].

## 2. Materials and Methods

The present analysis of AP firing and potential blockages of traveling AP waveforms is based on the original distributed HH cable model [26]. The numerical implementation was similar to that discussed in the literature [33,34,35,36,37]. Thus, the membrane potential of the HH neuron was given by the following formula:

(1) $$C_{m}\frac{dV\left(t\right)}{dt}=-G_{Na}\;m{\left(t\right)}^{3}\;h\left(t\right)\;\left[V\left(t\right)-E_{Na}\right]-G_{K}\;n{\left(t\right)}^{4}\;\left[V\left(t\right)-E_{k}\right]-G_{L}\;\left[V\left(t\right)-E_{L}\right]+I\left(t\right)\;\equiv -I_{ch}+I_{inj}\;$$

where *m*(*t*), *h*(*t*), and *n*(*t*) are the three gating variables. The first three terms on the right represent contributions to the ionic currents from the sodium, potassium, and leakage channels. These are denoted by *I_ch_*. The term *I*(*t*) represents any externally driven currents (=*I_inj_*) that might exist. In Equation (1), *V*(*t*) is the time-dependent membrane potential, while *G_K_*, *G_Na_*, and *G_L_* denote the potassium, sodium, and leakage conductance per unit area, respectively. In addition, *C_m_* represents the membrane capacitance per unit area, while *E_K_*, *E_Na_*, and *E_L_* are the corresponding reversal potentials. The accepted values for the various constants were chosen, with *C_m_* = 1 µF/cm^2^, *G_Na_* = 120 mS/cm^2^, *E_Na_* = 115 mV, *G_K_* = 36 mS/cm^2^, *E_K_* = 12 mV, *G_L_* = 0.3 mS/cm^2^, and *E_L_* = 10.6 mV. However, slight adjustments in the conductance and capacitance parameters are needed to adapt the HH model to myelinated nerves, namely, G_L_ = 0.015 mS/cm^2^ and *C_m_* = 0.0073 µF/cm^2^ [27]. The gating variables *m*(*t*), *n*(*t*)*,* and *h*(*t*) are taken to follow the well-known voltage-controlled, time-dependent Langevin equations [26] and are given in Equations (2a)–(2c) below for completeness:

(2a) $$\frac{dm\left(t\right)}{dt}=\alpha_{m}\;\left[V\left(t\right)\right]\;\left(1-m\left(t\right)\right)-\beta_{m}\;\left[V\left(t\right)\right]\;m(t)$$

(2b) $$\frac{dh\left(t\right)}{dt}=\alpha_{h}\;\left[V\left(t\right)\right]\;\left(1-h\left(t\right)\right)-\beta_{h}\left[V\left(t\right)\right]\;h(t)$$

(2c) $$\frac{dn\left(t\right)}{dt}=\alpha_{n}\;\left[V\left(t\right)\right]\;\left(1-n\left(t\right)\right)-\beta_{n}\left[V\left(t\right)\right]\;n(t)$$

The six voltage-dependent opening and closing rate functions *α_i_* and *β_i_* given in the equation set Equations (2a)–(2c) above are well-known and, based on experimental determinations, are given explicitly by the following expressions:

(3a) $$a_{m}\left[V\left(t\right)\right]=\frac{0.1\;(25-V\left(t\right))}{exp\left[\frac{25-V\left(t\right)}{10}\right]-1}$$

(3b) $$\beta_{m}\left[V\left(t\right)\right]=4\;exp\left[-\frac{V\left(t\right)}{18}\right]$$

(3c) $$a_{h}\left[V\left(t\right)\right]=0.07\;exp\left[-\frac{V\left(t\right)}{20}\right]$$

(3d) $$\beta_{h}\left[V\left(t\right)\right]=\frac{1}{\exp\left[\frac{30-V\left(t\right)}{10}\right]+1}$$

(3e) $$a_{n}\left[V\left(t\right)\right]=\frac{0.01\;(10-V\left(t\right))}{exp\left[\frac{10-V\left(t\right)}{10}\right]-1}$$

(3f) $$\beta_{n}\left[V\left(t\right)\right]=0.125\;exp\left[-\frac{V\left(t\right)}{80}\right]$$

The full set of equations given above was solved using a finite difference backward Euler scheme [38] to obtain the time-dependent action potentials and the gating functions.

**Figure 1:**
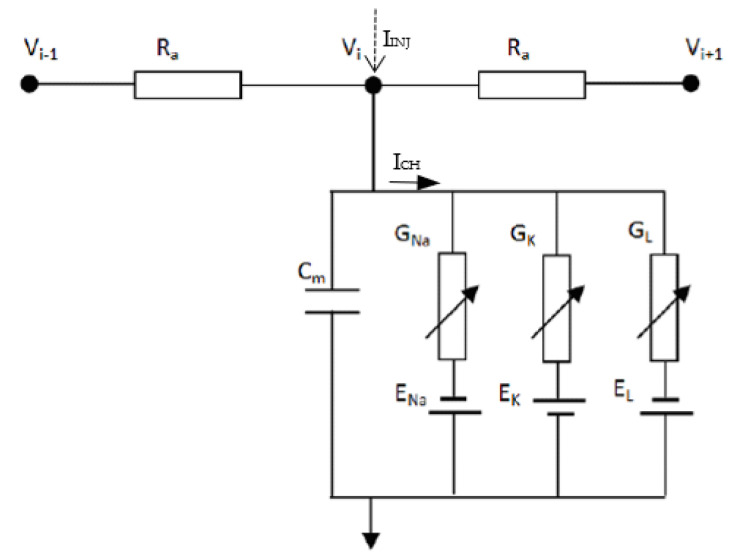
Equivalent circuit representing the cable line model for an unmyelinated nerve segment. The location and directions of the stimulus current (*I_INJ_*) and channel current (*I_CH_*) are indicated in the figure.

The entire nerve is considered to be spatially discretized as a one-dimensional distributed system, represented by nodes connected via axonal resistances. The related ionic channels are represented in the form shown in Equation (1). In the model representation shown in Figure 1, *R_a_* and *C_m_* represent the axonal resistance and membrane capacitance, respectively. Finally, *V_i_* represents the internal membrane potential at the node of interest, while *V_i_*_−1_ and *V_i_*_+1_ represent its neighboring nodes. The equation for AP propagation is given by

(4a) $$\frac{{V_{i-1}}^{k+1}-{V_{i}}^{k+1}}{R_{a}}-\frac{{V_{i}}^{k+1}-{V_{i+1}}^{k+1}}{R_{a}}+{I_{inj}}^{k+1}-{I_{ch}}^{k+1}=C_{m}{{\dot{V}}_{i}}^{k+1}$$

(4b) $$\frac{{V_{i-1}}^{k}-{V_{i}}^{k}}{R_{a}}-\frac{{V_{i}}^{k}-{V_{i+1}}^{k}}{R_{a}}+{I_{inj}}^{k}-{I_{ch}}^{k}=C_{m}{{\dot{V}}_{i}}^{k}$$

where *V_i_*_−1_*^k^*^+1^ represents the internal membrane potential at time step *k*+1, while *I_ch_* is the channel current, and *I_inj_* is the injected current pulse. Though *V_i_* refers to the internal node voltages, these are related to the transmembrane voltage V* by

(5) $$V_{i}=V_{e}+V_{rest}+V^{*}$$

where the resting potential *V_rest_* is a constant, while *V_e_* is the external potential taken to be zero (the reference ground potential). The channel current, *I_ch_*, which is a non-linear ionic current, has been given as part of Equation (1). The discretized version of the membrane voltages, based on a midpoint implicit scheme, evaluates to the following:

(6) $${V_{i}}^{k+1}={V_{i}}^{k}+\frac{\Delta t}{2}\left[{{\dot{V}}_{i}}^{k}+{{\dot{V}}_{i}}^{k+1}\right]$$

Finally, using an implicit scheme, the gating variables *m*(*t*), *n*(*t*), and *h*(*t*) can be similarly updated using numerical methods. This is shown below for *m*(*t*) as a specific example:

(7a) $$m_{i}^{k+1}=m_{i}^{k}+\frac{∆t}{2}\left[{\dot{m}}_{i}^{k}+{\dot{m}}_{i}^{k+1}\right]$$

(7b) $$m_{i}^{k+1}=\frac{m_{i}^{k}+\frac{∆t}{2}\left[{\alpha_{m,i}}^{k}+{\alpha_{m,i}}^{k+1}-{\alpha_{m,i}}^{k}{m_{i}}^{k}-{\beta_{m,i}}^{k}{m_{i}}^{k}\right]}{1+\frac{∆t}{2}\left[{\alpha_{m,i}}^{k+1}+{\beta_{m,i}}^{k+1}\right]}$$

## 3. Results and Discussion

**Figure 2:**
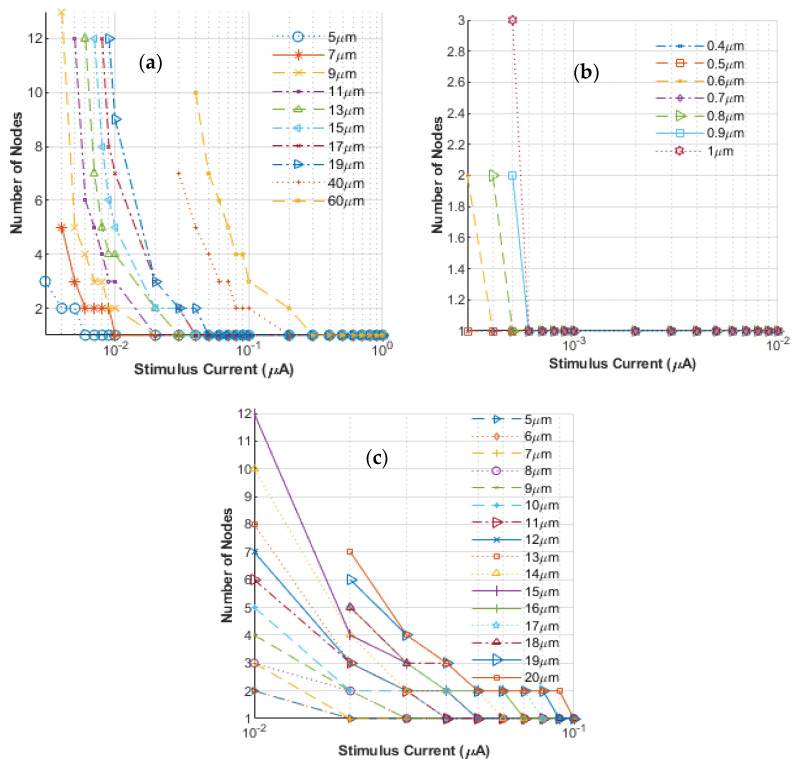
Stimulus current required for nerve activation as a function of the number of electrodes stimulated. (**a**) Results for general large cross-section unmyelinated nerves, (**b**) plot for very small cross-section unmyelinated nerve fibers, and (**c**) results for myelinated nerves of varying cross-sections.

Simulations for AP triggering were carried out based on the model presented in the previous section. The goal was to determine the activation current thresholds in an unmyelinated nerve and to numerically quantify any changes in the value brought about upon using multiple electrodes. To this end, nerves of different sizes, with radii ranging from 0.4 μm to 60 μm, were simulated. While unmyelinated nerves in humans are typically under 1 μm, this study explores the mechanics of nerve activation for unmyelinated nerves across species where nerves of significantly larger cross-sections exist. However, for the sake of clarity and completeness, we also provide a few results specific to the human nerve diameters. The results obtained are shown in Figure 2a–c with the number of stimulating nodes plotted against the threshold stimulus current required to just launch an AP. For convenience, a semi-logarithmic scale was chosen, since the threshold currents varied over a considerable range. In all cases simulated, the stimulus was taken to be a DC pulse that had a fixed 1 ms duration. The time for current injection was kept fixed for consistency across each run for the various cases of differing parameters, such as nerve diameters, the number of stimulating nodes used, and the stimulus amplitude chosen for the different simulations. Each node in the plot represents a 1 mm region The features evident from the curves shown in Figure 2a–c are as follows: (i). In general, as perhaps might be expected, the injection current needed to initiate an AP decreases as the number of stimulating nodes increases. (ii) Since the number of stimulating nodes is an integer variable, the curves are discrete and exhibit step-like behaviors. The curves eventually flatten out when the situation comes down to just one stimulating node, and the least current value at the verge of this flattening should be taken as the true indicator of the current threshold. (iii) The triggering current needed to initiate an AP is seen to depend on the nerve size, with smaller nerves requiring lower stimulating excitation. Again, this is in line with qualitative expectations. (iv) Finally, even though one can try to increase the number of electrodes that might stimulate the neural system, the injection current required does not scale. In fact, it is predicted to diminish ever so slowly with an increasing number of triggering points. Thus, the benefit of requiring an ever-smaller current trigger is seen to reach a limit, with a lower bound dependent on the neural fiber.

**Figure 3:**
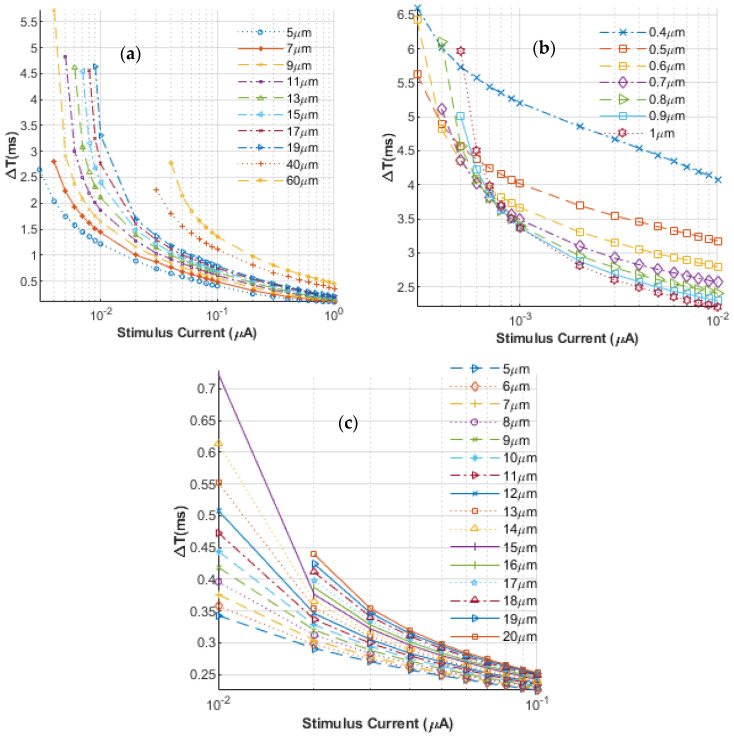
Simulation results showing the delay between the application of the stimulating currents and the launch of an AP for different nerve sizes: (**a**) for nerves with radii greater than 1 μm, (**b**) for nerves with radii less than 2 μm, and (**c**) for myelinated nerves.

Next, the delay between the application of the stimulating currents and the launch of an AP was evaluated, and the results obtained are shown in Figure 3 for both unmyelinated and myelinated nerves. The plot in Figure 3a represents nerves with radii greater than 1 μm, while the results in Figure 3b are given for nerves with radii less than 2 μm. Figure 3c shows simulated data for myelinated nerves. The stimulus in each case was again taken to be a DC pulse with a fixed 1 ms duration. The plot shows the delay time in milliseconds as a function of the stimulating current, *I_inj_*. The values of *I_inj_* are seen to range from ~3 nA to about 1 μA, while the corresponding delay times range from less than 0.1 ms to more than 5.5 ms. Again, as might be expected, the results indicate shorter AP turn-on times at higher currents. The neuron sizes for this set of simulations ranged from 5 μm to 60 μm, consistent with previous studies. Combining the results of Figure 1 and Figure 2 can then provide a complete picture of the expected time delay for an AP launch, given a stimulating current and the number of electrodes used for a given nerve radius.

**Figure 4:**
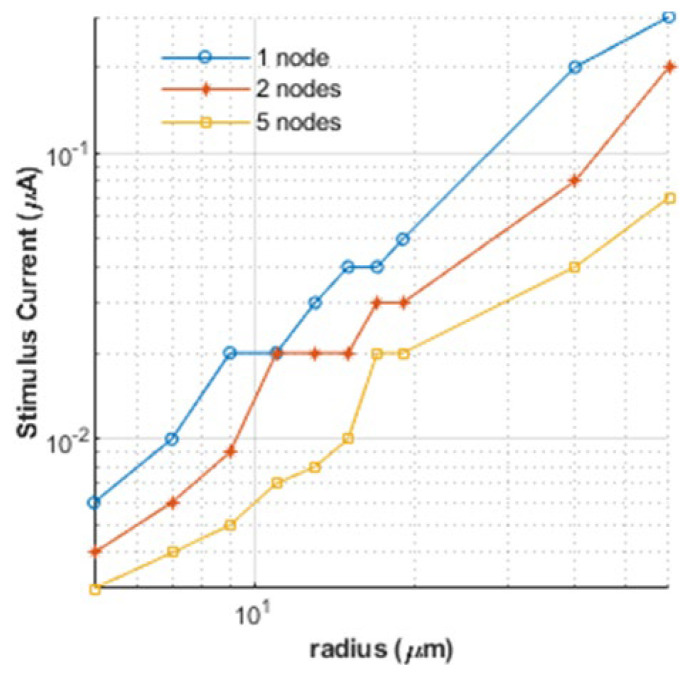
Minimum stimuli required for successful nerve activation when using one or more stimuli for nerves with different radii.

For completeness, the numerical predictions of changes in the stimulus current with nerve radii are given in Figure 4. The non-linear relationship that is inherent from Equation (1) between the nerve radius and the successful activation (determined here by a minimum transmembrane potential of 80 mV) required that logarithmic scales be used on both axes in Figure 4 to produce sufficient visual fidelity. Due to the discreteness of the stimulating node numbers that go into the calculation, the plots are not smooth. Figure 4 shows the results for three different numbers of stimulating electrodes. In general, the highest value of *I_inj_* is predicted for the stimulation of the least number of nodes for any given size. In addition, as expected, the requisite current increases as the nerve radius increases in dimension.

**Figure 5:**
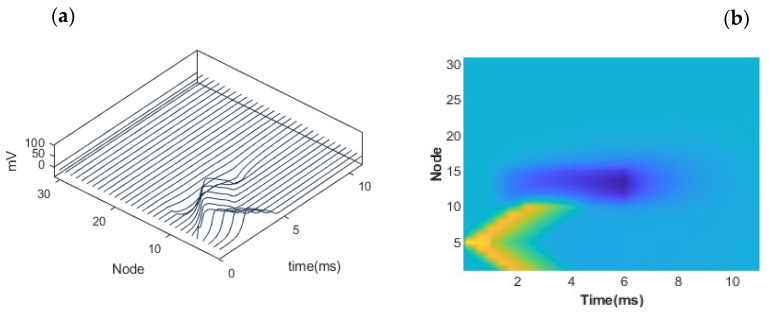
Simulation of an action potential block. A nerve segment stimulated near one of its ends results in a successful interruption when a suitable DC pulse is applied. (**a**) Three-dimensional results of the propagating voltage wave versus position in terms of node numbers and time. (**b**) A two-dimensional view showing the successful interruption of the upper branch with an interruption signal applied at node 11.

Having discussed the results for the AP initiation of nerves of various sizes, stimulated by different numbers of current injecting electrodes, the next set of results focused on blocking a propagating AP in an unmyelinated nerve. This was accomplished by first launching an AP from some point within the one-dimensional distributed unmyelinated nerve system based on an injecting current trigger. Next, a second interruption pulse was applied at a location downstream. In the results shown in Figure 5a,b, the separation between the initiation point and the interruption signal was taken to be 6 mm, while the nerve radius was 12.5 μm. Figure 5a reveals a three-dimensional result of the propagating voltage wave versus position (in terms of integral node numbers) and time. It shows the launch of an AP wave (from node number five), which starts propagating bilaterally in two opposite directions. The voltage values of the propagating AP wave are included in the figure. The interruption pulse was applied at node eleven. The result in Figure 5a shows the AP coming to a stop as it passes the interruption point. A better visualization is seen in Figure 5b which is a two-dimensional view. The upper branch of the symmetric and bilateral AP wave launched is seen to stop upon reaching the interruption location.

**Figure 6:**
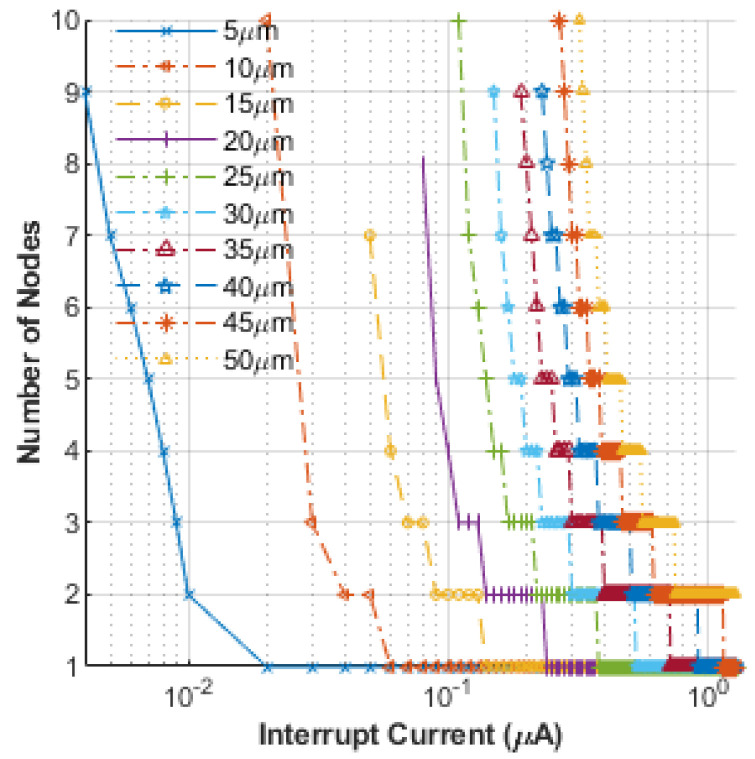
Interrupting the AP propagation in a nerve segment by applying lower interruption signals over multiple nodes.

Next, the possible effects of applying interruption signals over multiple nodes of a nerve segment were probed to evaluate any possible reductions in the requisite interruption amplitude. In these simulations, all interruption signals were taken to be identical and concurrent, i.e., they all were assigned the same amplitude, time duration, and step function waveform. Thus, the interruption zone was expanded over larger regions to evaluate any possible reductions in the intensity of the interruption amplitude. The results obtained are shown in Figure 6. Each node in the plot represents a 1 mm region. The predictions shown are for varying nerve radii ranging from 5 μm to 50 μm. Given the large range of stimulus currents (0.001 µA~5 µA), a semi-logarithmic scale is used for the plot. The results reveal that the interruption current required to achieve an AP block can be reduced as the number of nodes over which the interruption signal is applied is expanded. As may be expected, the smaller-sized nerves require the smallest interruption signal intensity. For computation purposes, a nerve segment was considered to block an incident AP if the transmembrane potential did not exceed 20 mV for any node in the interruption zone or any region further downstream. In addition, it was set up such that the interruption signal (linked to the stimulus signal) started as soon as the upstream excitation pulse ended, and it remained in effect till the end of the simulation. This result is important and would have practical relevance since it points to the possibility of arresting APs with a much lower blocking stimulus intensity by using multiple nodes. The lower intensity signals are indicative of a safer and more reliable approach to induce AP blockage.

**Figure 7:**
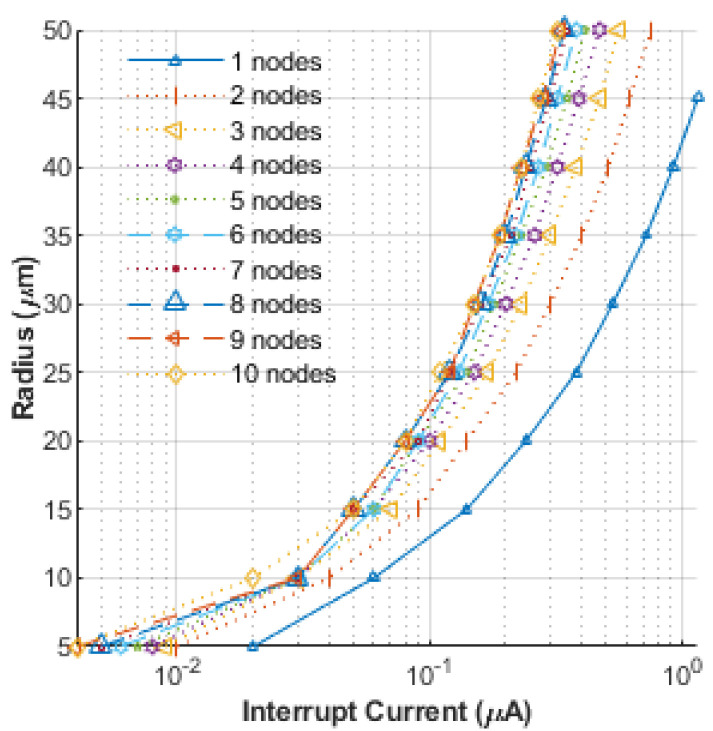
Relation between the nerve radius and the interruption current intensity with the width of interruption signal zone set as the parameter.

Finally, the relation between nerve radius and the interruption current signal is shown in Figure 7, with the width of the interruption signal zone taken as a parameter. For a given nerve size, the interruption current magnitude needed is predicted to decrease as the number of nodes over which the signal is applied increases. For the application of the interruption at multiple nodes, all signals were taken to have identical amplitudes, durations, and the same step-pulse waveforms. In addition, as the nerve radius decreases, the required interruption signal magnitude is predicted to go down. Such AP blockage in nerves has applications ranging from neurophysiology, clinical research, neuromuscular stimulation therapy, to muscular and sensory incapacitation.

Finally, it may be mentioned that the present study does not explore aspects such as noise in neural circuits or related aspects such as possible stochastic resonance phenomena [39,40,41]. These are aspects that could be incorporated and studied through numerical simulations in the future.

## 4. Conclusions

Systems and devices based on electrical stimulation are commonly used for a large variety of therapeutic bio-applications. Unlike chemical or pharmacological methods for medical treatments, electrical means offer benefits that include speed, selectivity and control, and more direct pathways for remedial action. Many of the applications, such as neuromuscular control by artificial electrical stimulation, require the use of multielectrode systems to selectively activate a group of nerves. Multielectrode systems are also important for the realization of neuro-prosthetic systems. Therefore, given the need for multielectrode stimulation devices in many practical applications, we have undertaken a study to probe some of the details of nerve activation resulting from multielectrode excitation. In particular, aspects such as the magnitudes of the threshold stimulus current, its variation with the number of electrodes used, the effect on nerves of different radii, and the possibility of blocking an AP have been quantitatively analyzed. This constitutes a simple first step towards assessing neural responses and other potential bioeffects produced by multielectrode stimulation, with eventual progression towards tissue and whole-body assessments. To the best of our knowledge, such aspects have not been examined.

The injection current needed to initiate an AP is predicted to decrease as the number of stimulated nodes increases. The actual threshold current does depend on the nerve size, with smaller nerves requiring lower stimulating excitation. It has also been shown that the injection current required to launch an AP diminishes slowly with an ever-increasing number of triggering electrodes. Furthermore, the time delays in launching an AP were shown to decrease with increasing stimulation current. Finally, it was demonstrated that arresting the AP propagation in a nerve segment could be achieved by applying lower magnitudes of the interruption signal if more nodes of a multielectrode array were to be used. This result is important and would have practical relevance, since the lower intensity signals for interrupting AP propagation indicate a safer and more reliable approach to AP blockage. From a practical standpoint, the complexity should not increase appreciably, as multielectrode systems have been proposed and even demonstrated in the literature [42,43,44].

## Data Availability

The data presented in this study are available on request from the corresponding author due to privacy restrictions.
